# Asymmetrical Relationship between Prediction and Control during Visuomotor Adaptation

**DOI:** 10.1523/ENEURO.0280-18.2018

**Published:** 2018-12-14

**Authors:** James Mathew, Pierre-Michel Bernier, Frederic R. Danion

**Affiliations:** 1 Aix Marseille Université, CNRS, Institut de Neurosciences de la Timone UMR 7289, 13005 Marseille, France; 2 Département de Kinanthropologie, Université de Sherbrooke, Sherbrooke, Québec J1K 2R1, Canada

**Keywords:** human, internal model, learning, motor control, prediction, transfer, eye-hand coordination

## Abstract

Current theories suggest that the ability to control the body and to predict its associated sensory consequences is key for skilled motor behavior. It is also suggested that these abilities need to be updated when the mapping between motor commands and sensory consequences is altered. Here we challenge this view by investigating the transfer of adaptation to rotated visual feedback between one task in which human participants had to control a cursor with their hand in order to track a moving target, and another in which they had to predict with their eyes the visual consequences of their hand movement on the cursor. Hand and eye tracking performances were evaluated respectively through cursor–target and eye–cursor distance. Results reveal a striking dissociation: although prior adaptation of hand tracking greatly facilitates eye tracking, the adaptation of eye tracking does not transfer to hand tracking. We conclude that although the update of control is associated with the update of prediction, prediction can be updated independently of control. To account for this pattern of results, we propose that task demands mediate the update of prediction and control. Although a joint update of prediction and control seemed mandatory for success in our hand tracking task, the update of control was only facultative for success in our eye tracking task. More generally, those results promote the view that prediction and control are mediated by separate neural processes and suggest that people can learn to predict movement consequences without necessarily promoting their ability to control these movements.

## Visual Abstract

**Figure:**
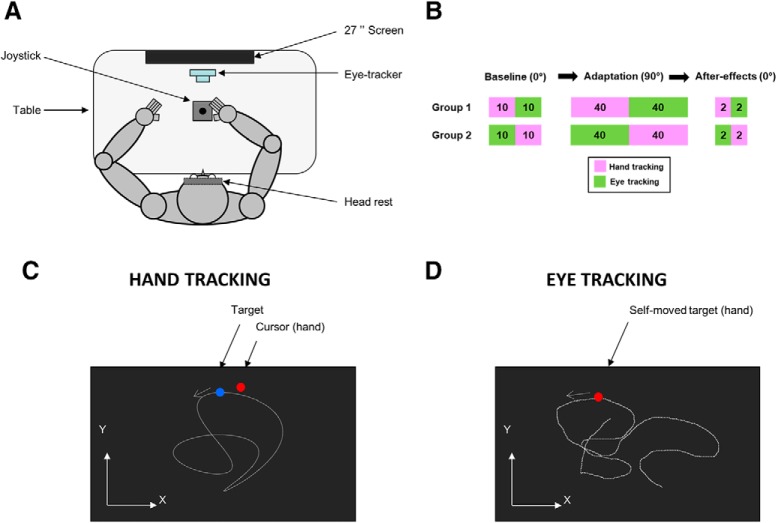

## Significance Statement

Current theories suggest that accurately predicting the sensory consequences of one’s actions is central for perception, awareness of action, and motor learning. In the latter case, it is assumed that prediction errors are used to train the controller that transforms our desired sensory consequences into motor commands. Here we show that, following exposure to biased hand visual feedback, people can update their ability to predict visual consequences of hand movements without necessarily improving their ability to control these movements. This work challenges the view that the joint update of prediction and control is mandatory when facing a change in the mapping between motor commands and sensory consequences. Instead, we propose that task demands mediate the update of prediction and control.

## Introduction

Current theories suggest that skilled motor behavior depends on the ability to control our body and to predict the consequences of this control (Flanagan et al., 2003; Shadmehr et al., 2010; Shadmehr, 2017). Whereas prediction is used to transform motor commands into expected sensory consequences, control is used to transform desired sensory consequences into motor commands (Kawato, 1999). In the internal model approach, the former mechanism is accounted for by a forward model, also called the predictor or state estimator (Miall and Wolpert, 1996; Wolpert and Ghahramani, 2000; Wolpert and Flanagan, 2001; Todorov, 2004); and the second one is accounted for by an inverse model, also called the controller (Todorov, 2004; Shadmehr et al., 2010) or control policy (Diedrichsen et al., 2010; Scott, 2012). When the mapping between a motor command and its sensory consequences is altered by a change in the body or the environment, forward and inverse models may operate independently of each other or in tandem to preserve accurate performance (Wolpert and Kawato, 1998; Wolpert and Ghahramani, 2000; Haruno et al., 2001; Honda et al., 2018). Although technically challenging (Lalazar and Vaadia, 2008; Mulliken et al., 2008), experimental evidence for separate processes underlying prediction and control are scarce (Flanagan et al., 2003; Honda et al., 2018). In an elegant study, Flanagan et al. (2003) showed that anticipatory grip force adjustments were updated before participants learned to adequately control the trajectory of a grasped object with unusual dynamics. They interpreted this result as evidence that the update of the predictor (forward model) precedes the update of the controller (inverse model). Not only is this finding consistent with the idea that updating a forward model is computationally simpler (Jordan and Rumelhart, 1992; Wolpert and Kawato, 1998), but it also supports the view that the forward model plays a role in training the inverse model (Bhushan and Shadmehr, 1999; Haruno et al., 2001; Haith and Krakauer, 2013). However, to our knowledge, direct evidence for a causal relationship between the update of prediction and control is still lacking. In particular, it remains unknown whether it is possible to update prediction without promoting the update of control, and vice versa.

The goal of this study was to investigate the degree of coupling between the update of prediction and control during sensorimotor adaptation. To address this issue, we compared two situations in which participants had to adapt to visuomotor rotation, a paradigm in which the visual feedback of the hand is rotated (Krakauer, 2009). In the first situation, participants were required to manually control a cursor so as to track a visual target following a smooth but unpredictable trajectory (Tong and Flanagan, 2003; Ogawa and Imamizu, 2013). In this case, we reasoned that an update of both the inverse and forward models would be beneficial for task success. Indeed, not only does it appear necessary to update the mapping between a desired cursor position and hand motor commands, but it is advocated that this update of the inverse model is guided by the forward model, which itself is updated based on prediction errors (Haith and Krakauer, 2013). Conversely, computational modeling shows that updating the forward model in isolation of the inverse model is insufficient to produce optimal movements (Aprasoff and Donchin, 2012; Honda et al., 2018). In the second situation, participants exposed to the same visuomotor rotation were required to track with their eyes a visual target that was self-moved via random hand motions (i.e., with no explicit spatial goal), a task used to probe the ability to predict the visual consequences of one’s own movement (Vercher et al., 1995; Landelle et al., 2016) by means of a forward model of the arm (Vercher et al., 2003). As a result, although in this case the update of the forward model of the hand seems mandatory for accurate eye tracking, updating the inverse model of the hand is not obligatory because producing spatially accurate hand movements is not a task requirement.

To determine how strongly coupled the update of control and prediction are, we investigated the transfer of adaptation between these two tasks, and explicitly asked whether prior adaptation of hand tracking facilitates the adaptation of eye tracking, and vice versa. If the update of control and prediction remain coupled irrespective of tasks, we expect large and reciprocal transfer of adaptation across the two tasks. In contrast, if a joint update is only necessary for hand tracking, we expect an asymmetrical transfer such that hand tracking benefits eye tracking, but not the other way around.

## Materials and Methods

### Participants

Twenty-four healthy right-handed volunteers (mean ± SD age, 27.2 ± 6.9 years; 16 females) were recruited. The experimental paradigm (2016-02-03-007) was approved by the local ethics committee of Aix-Marseille university and complied with the Declaration of Helsinki. All participants gave written consent before participation.

### Apparatus

**Figure 1.**
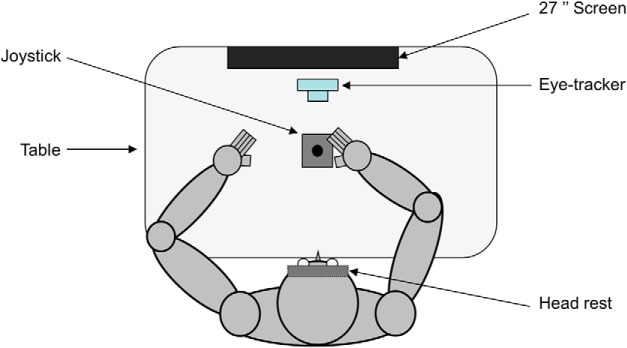
Schematic view of the apparatus. Top view of a participant sitting in the experimental setup (see Materials and Methods for further information).

Figure 1 shows the experimental setup. Participants were seated comfortably in a dark room facing a screen (BENQ; resolution, 1920 × 1080; 27 inches; 144 Hz) positioned on the frontal plane 57 cm away from participants eyes (1 cm on the screen = 1° of visual angle). Participants’ head movements were restrained by a chin rest and a padded forehead rest so that the eyes in primary position were directed toward the center of the screen to block vision of their hands, a piece of cardboard was positioned under the participants’ chins. They were required to hold with the right hand a joystick (with ±25° of inclination along the *x*- and *y*-axes; Serie 812, Megatron) positioned horizontally on a table in front of them, along the sagittal plane. Note that there was no assistive force to bring back the joystick to the central position. Both right and left forearms were resting on the table. The output of the joystick was fed into a data acquisition system (Keithley ADwin Real Time, Tektronix) and sampled at 1000 Hz. Eye movements were recorded using an infrared video-based eye tracker (Eyelink desktop-mounted system, SR Research). Horizontal and vertical positions of the right eye were recorded at a sampling rate of 1000 Hz. The output from the eye tracker was calibrated before every block of trials by recording the raw eye positions as participants fixated a grid composed of nine known locations. The mean values during 1000 ms fixation intervals at each location were then used off-line for converting raw eye tracker values to horizontal and vertical eye position in degrees of visual angles.

### Procedure

**Figure 2.**
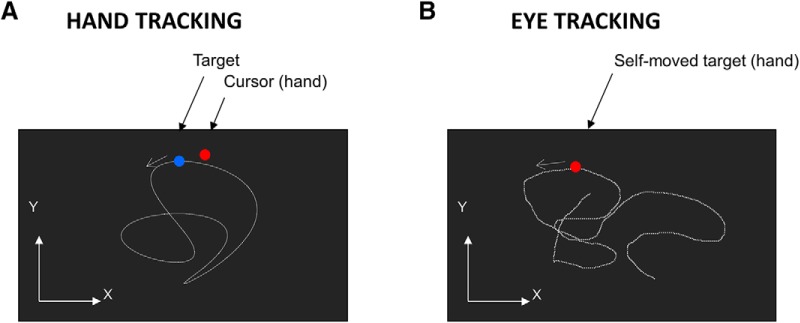
Experimental tasks. ***A***, Schematic view of the screen in the hand tracking condition. ***B***, Schematic view of the screen in the eye tracking condition (see Materials and Methods for further information).

Throughout the main experiment, participants performed two types of tracking tasks. During the hand tracking task (Fig. 2*A*), participants had to move the joystick with their right hand so as to bring a cursor (red disk, 0.5° in diameter) as close as possible from a moving target (blue disk, 0.5° in diameter). This task was designed to probe the ability to produce hand movements along a desired trajectory (Tong and Flanagan, 2003; Ogawa and Imamizu, 2013).

The motion of the target resulted from the combination of several sinusoids: two along the frontal axis (one fundamental and a second or third harmonic), and two on the sagittal axis (same procedure). The following equations were used to construct target motion:

$$x_{t}=A_{1x}cos\omega t+A_{2x}cos(h_{x}\omega t-\varphi_{x})$$

$$y_{t}=A_{1y}\sin\omega t+A_{2y}sin(h_{y}\omega t-\varphi_{y})$$

**Figure 3.**
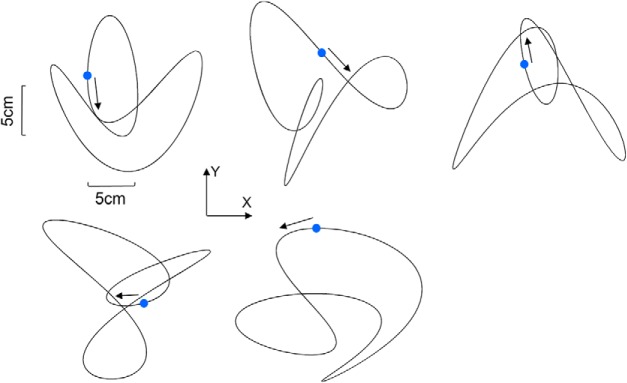
Target paths used across all experimental conditions. The blue dot shows the initial position of the target, and the arrow shows its initial direction. The paths are shown in the vertical plane (see Materials and Methods for more details).

This technique was used so as to generate pseudo-random 2D pattern while preserving smooth changes in velocity and direction (Mrotek and Soechting, 2007; Soechting et al., 2010). A total of five different patterns were used throughout the experiment (Table 1, Fig. 3). All target paths had similar lengths (160 cm). The order of patterns was randomized across trials while making sure that each block contained a similar number of each pattern. During this task, participants did not receive any explicit constraints regarding their gaze, meaning that they were free to look at the target, the cursor, or both.

**Table 1:** Target trajectory parameters

Trajectory	*A1x* (cm)	*A2x* (cm)	Harmonic *x*	Phase *x* (°)	*A1y* (cm)	*A2y* (cm)	Harmonic y	Phase *y* (°)
1	5	5	2	45	5	5	3	−135
2	4	5	2	−60	3	5	3	−135
3	4	5.1	3	−60	4	5.2	2	−135
4	5	5	3	90	3.4	5	2	45
5	5.1	5.2	2	−90	4	5	3	22.5

During the eye tracking task, participants were instructed to wiggle a red target (0.5° in diameter) on the screen by means of the joystick held in their right hand while concurrently keeping their eyes as close as possible from the self-moved target (Fig. 2*B*). This task was designed to probe the ability to predict the visual consequences of one’s hand movement (Vercher et al., 1995; Chen et al., 2016; Danion et al., 2017). Participants were asked to generate random target movements so as to make target motion as unpredictable as possible (Steinbach and Held, 1968; Landelle et al., 2016; Mathew et al., 2017). However, to maintain consistency across subjects and trials, we ensured that, over each trial, joystick movement led to a mean tangential target velocity close to 16°/s (thereby maintaining task difficulty relatively unchanged). Note that the mapping between hand and cursor tangential velocities was unaffected by the visuomotor rotation. To facilitate the production of random movements, a template was provided on the screen during demonstration trials. In addition, during the experimental trials, mean target velocity was computed on-line so that experimenters could provide verbal feedback to the participants such as “please move faster” or “please slow down” when necessary. This procedure ensured minimal differences in mean target velocity across participants (SD = 1°/s) and trials (SD = 0.66°/s). Note that participants were encouraged to cover the whole extent of the screen, but the gain of the joystick (25° inclination = 15 cm on the screen) was adjusted to prevent excursion of the target outside of the screen. Thanks to this procedure, corrections in hand movements were unnecessary to keep the cursor within the screen.

For both eye and hand tracking, the task could be performed under a simple and a complex mapping. Under the simple mapping, the relation between the joystick motion and its visual consequences on the screen was very intuitive, mimicking the behavior of a computer mouse. This mapping was intended to provide baseline tracking performance. Under the complex mapping, the previous mapping was rotated counterclockwise by 90° (Ogawa and Imamizu, 2013). This unusual mapping was intended to elicit adaptation. Note that, however, this visuomotor rotation does not alter the mapping between hand motor command and tangential velocity of hand movement consequences on the screen. The duration of a trial was 10 s for both the eye- and hand tracking tasks.

**Figure 4.**
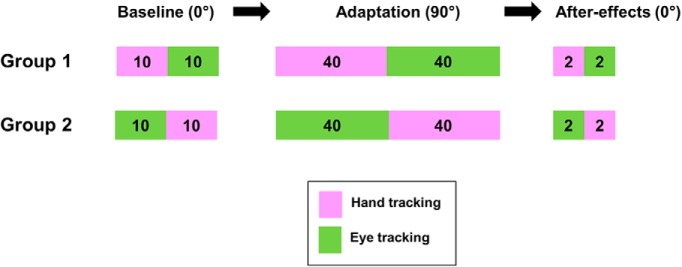
Experimental design for each group of participants (see Materials and Methods for further information).

As shown in Figure 4, participants were split into two groups that both practiced the eye and hand tracking tasks, under the simple and the complex mapping, albeit in different orders. The experimental session consisted of three phases. During the initial phase (baseline, 0°), the first group of participants (*N* = 12) performed one block of 10 trials of hand tracking followed by one block of 10 trials of eye tracking. Subsequently during the adaptation phase (90°), this group performed one block of 40 trials of the hand tracking task followed by one block of 40 trials of the eye tracking task, both under the rotated mapping. During the final phase, the initial mapping was unexpectedly restored (0°), allowing to test for aftereffects with two trials of hand tracking followed by two trials of eye tracking. The second group of participants followed the same protocol (baseline, adaptation, and aftereffects), but, for each phase, the order of eye and hand blocks was reversed. This experimental design was selected to assess the possible transfer of learning between our two tracking tasks by means of group comparisons (Danion et al., 2012).

### Control experiment

To demonstrate the involvement of predictive mechanisms in our eye tracking task, we also performed a control experiment with 10 right-handed new participants (mean age, 28.6 ± 7.3 years; 5 females) in which we compared eye tracking performance with a self-moved target versus an externally moved one. Practically, each participant completed one block of 10 trials using the original version of the eye tracking task (self-moved target, no rotation), followed by a block of 10 trials in which participants had to track with the eyes the target trajectories that they had produced during the first block (Landelle et al., 2016).

### Data analysis

To assess hand tracking performance, we measured the mean Euclidian distance between the cursor (moved by hand) and the externally moved target for each trial. For eye tracking, we measured the mean Euclidian distance between the eye and the self-moved target. To gain more insight about gaze behavior during hand tracking, we also measured the mean Euclidian distance between eye and target, as well as between eye and cursor. Our motivation was to assess whether gaze tracked more closely the cursor or the target. For all these computations, the first second of each trial was discarded. Furthermore, all eye, cursor, and target *x* and *y* signals were separately low-pass filtered with a Butterworth filter (4th order) using a cutoff frequency of 25 Hz. Note that, based on the pupil diameter (which was also recorded), blinks were removed from our eye recordings (∼0.9%). The temporal relationship between eye and target was estimated by means of cross-correlations that simultaneously took into account the vertical and horizontal axes. To simultaneously cross-correlate horizontal (*x*) and vertical (*y*) position signals between effectors, we interleaved the *x* and *y* signals and always time shifted these interleaved signals by a multiple of two samples (Flanagan et al., 2008; Danion and Flanagan, 2018). Further analyses showed that this method led to results similar to those obtained by averaging the lags provided by cross-correlations conducted separately for *x* and *y* signals.

To provide further information about gaze behavior, we also assessed the characteristics of catch-up saccade (Steinbach and Held, 1968; Mathew et al., 2017). The identification of catch-up saccades required computing the tangential velocity and acceleration of the eye. First, *x* and *y* eye position signals were differentiated to obtain the velocity traces. Then the eye velocity signals were low-pass filtered with a cutoff frequency of 25 Hz to remove the noise from the numerical differentiation. The resultant tangential eye velocity was then differentiated to provide the tangential acceleration that we also low-pass filtered at 25 Hz to remove the noise. Saccades were identified based on the acceleration and deceleration peaks of the eye (>1500°/s^2^). Further visual inspection allowed the identification of smaller saccades (<1°) that could not be identified automatically by our program. Following the identification of catch-up saccades for each trial, we computed their average number of saccades per second (saccade rate) as well as their mean amplitude; again, the first second of each trial was excluded.

To assess the randomness of hand motion during eye tracking, approximate entropy (ApEn) was used as an index that characterizes the unpredictability of a signal (Pincus, 1991); the larger the ApEn, the more unpredictable the signal is. To compute ApEn, we used the following Matlab code: https://fr.mathworks.com/matlabcentral/fileexchange/32427-fast-approximate-entropy [with the following settings: embedded dimension = 2, tolerance = 0.2 × SD (target trajectory)]. Note that ApEn was computed separately for the *x* and *y* components of the joystick motion.

### Statistics

Two-way ANOVAs were used to assess the effects of GROUP (i.e., with/without prior experience) and trial rank (TIME). The Newman–Keuls technique was used for *post hoc* tests to correct for multiple comparisons. A logarithmic (*z* score) transformation was used to normalize the distribution of *R* values. A 0.05 significance threshold was used for all analyses.

## Results

### Representative trials

**Figure 5.**
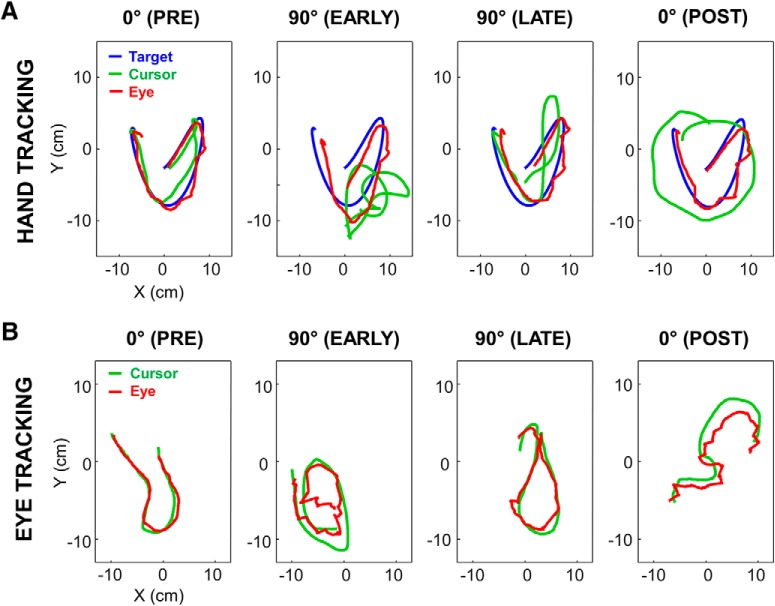
Typical trials under each experimental condition at various moments. ***A***, Target, cursor, and eye position signals in the hand tracking task, during pre-exposure, early exposure, late exposure, and after exposure to the 90° visuomotor rotation. ***B***, Same as ***A*** for eye and cursor position signals in the eye tracking task. Trials presented on the top and bottom rows were performed by two distinct participants. Although each trial was 10 s long, only 2.5 s of signals are displayed for clarity.

Figure 5 plots representative trials collected from two naive participants in each task at various stages of exposure. As can be seen, in both tasks, tracking performance was substantially altered immediately after the introduction of the visuomotor rotation (PRE vs EARLY). However, for both tasks tracking performance improved across trials as suggested by the comparison between EARLY and LATE trials. When the rotation was unexpectedly removed (POST), tracking performance was altered, demonstrating the presence of sensorimotor adaptation (i.e., aftereffects). In the next sections, we analyze in more detail the time course of this adaptation to the rotation and assess whether adaptation in a given task was facilitated by prior experience in the other task.

### Adaptation and transfer of adaptation

**Figure 6.**
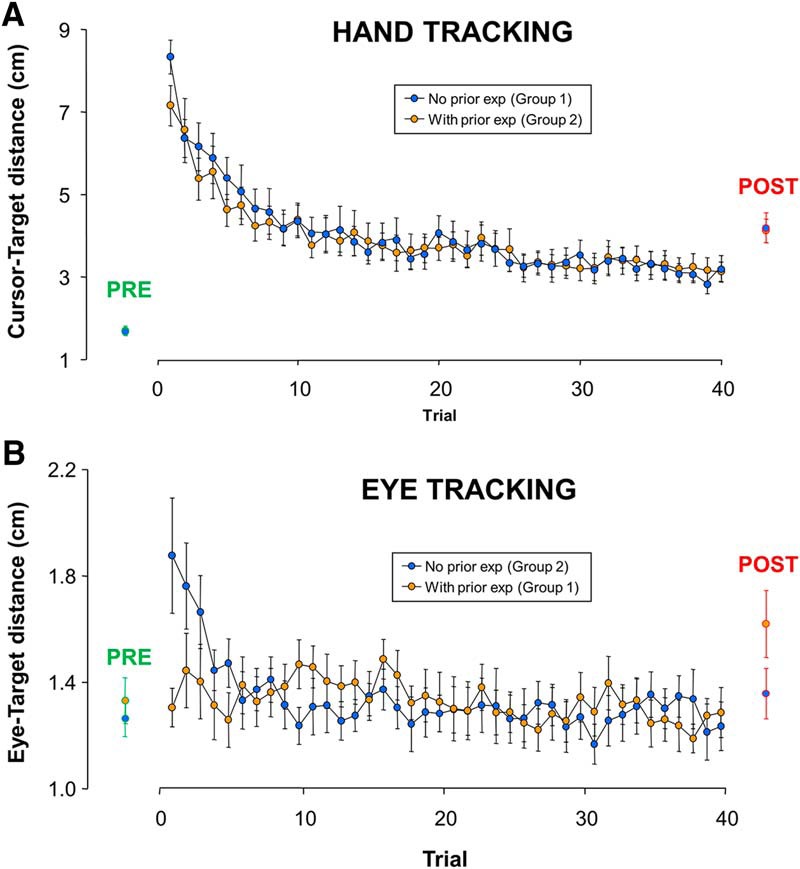
Time course of adaptation in each task as a function of prior experience in the other task. ***A***, Comparison of hand tracking performance with and without prior eye tracking experience. ***B***, Comparison of eye tracking performance with and without prior hand tracking experience. Although prior eye tracking experience did not influence hand tracking adaptation, prior hand tracking experience markedly facilitated eye tracking adaptation. Error bars represent the SEM.

In Figure 6, we present the mean tracking performance across trials in each group separately for the hand-tracking task (Fig. 6*A*) and the eye tracking task (Fig. 6*B*). We first address the effect of the visuomotor rotation on naive participants (Fig. 6*A*,*B*, blue dots; i.e., participants that did not receive prior exposure to the perturbation). When first exposed to the visuomotor rotation, performance in both hand and eye tracking was severely altered. Indeed, the cursor–target distance in the hand-tracking task (first two trials) increased by 345% with respect to baseline (last two trials). Similarly, the eye–target distance in the eye tracking task (first two trials) increased by 50% with respect to baseline (last two trials). As expected, tracking performance improved across trials, but learning curves exhibited different dynamics across tasks. Indeed, hand tracking performance never returned to baseline, even after the 40th trial, whereas eye tracking performance returned to baseline around the 10th trial. When the rotation was unexpectedly removed aftereffects were observed in both cases (see later section on aftereffects), thereby confirming the adaptation of an internal model accounting for the mapping between hand motor commands and visual consequences.

We next address our main issue, namely whether prior adaptation to the visuomotor rotation during eye tracking benefited hand tracking, and vice versa. We first focus on the hand tracking task (Fig. 6*A*) by comparing the learning curve exhibited by naive participants (Fig. 6*A*, in blue) to that of participants having already adapted to the eye-tracking task (Fig. 6*A*, in orange). A conventional two-way ANOVA was conducted comparing early (1–2), intermediate (19–20), and late (39–40) trials across the two groups. Not surprisingly, the effect of TRIAL was significant (*F*_(2,44)_ = 152.70; *p* < 0.001). Most importantly, however, there was no main effect of GROUP (*F*_(1,22)_ = 0.12; *p* = 0.72), as well as no interaction between TRIAL and GROUP (*F*_(2,44)_ = 0.79; *p* = 0.46), suggesting similar learning curves. To further investigate this issue, we extracted exponential fitting parameters ($Error=ae^{b\times trial}+c$) for each participant (individual *R* values ranging from 0.65 to 0.98; *p* < 0.001). For each of the three fitting parameters, the ANOVA showed no significant effect across groups. Namely, the initial performance (parameter a; *F*_(1,22)_ = 2.62; *p* = 0.12), the learning rate (parameter b; *F*_(1,22)_ = 0.45; *p* = 0.51), and the asymptote (parameter c; *F*_(1,22)_ = 0.07; *p* = 0.78) were similar across groups. Overall, we found no evidence for a transfer of adaptation from eye to hand tracking, suggesting that prior adaptation to the visuomotor rotation though eye tracking does not benefit hand tracking.

In contrast, prior adaptation of hand tracking appeared to markedly facilitate adaptation of eye tracking. Indeed, as evidenced in Figure 6*B*, there was a clear difference in initial performance between the naive and the experienced group. A key observation is that the group that previously adapted to the rotation during hand tracking showed no initial alteration in eye tracking performance. In support, two-way ANOVA comparing eye–target distance among early (1–2), intermediate (19–20), and late (39–40) trial pairs across the two groups showed a significant interaction between TRIAL and GROUP (*F*_(2,44)_ = 8.36; *p* < 0.001). Breakdown of the interaction revealed that the initial trial pair of naive participants (1.82 cm) was greater than any of the five other trial pairs, none of which differed from baseline (1.30 cm). To further explore this issue, we used our exponential regression technique over the first 10 trials. For the naive group, individual regressions were significant for 10 of the 12 participants (*R* = 0.76 ± 0.07; *p* = 0.01). In contrast for the group that received prior exposure, none of the individual regressions was significant (*R* = 0.14 ± 0.04; *p* > 0.05). A similar contrast across groups was found when we examined the temporal relationship between the eye and the cursor motion. Two-way ANOVA comparing early (1–2) and late (39–40) trials across the two groups showed a significant interaction between TRIAL and GROUP (*F*_(1,22)_ = 5.39; *p* < 0.05). *Post hoc* analysis of the interaction showed that during early trials, this lag was greater for naive participants than for participants with prior training (88 vs 64 ms; *p* < 0.05). In contrast, during late trials the eye–target lag was similar for both groups of participants (55 vs 52 ms; *p* = 0.71). Overall, these analyses show that that prior adaptation of hand tracking strongly improved eye tracking, leading in fact to nearly complete transfer of adaptation from hand to eye tracking.

### After-effects

After-effects are crucial to assess the presence of sensorimotor adaptation. For both tasks, visual inspection of the right side of Figure 5, as well as the mean group data presented on the right side of Figure 6 (see red circles, POST), indicates the presence of after-effects when the visuomotor rotation was unexpectedly removed. This scheme was confirmed by two-way ANOVA comparing late trials (39–40) and post trials. During the hand-tracking task, there was a main effect of TRIAL (*F*_(1,22)_ = 8.726; *p* < 0.001), consistent with an increase in error (+34%). However, there was no main effect of GROUP (*F*_(1,22)_ = 0.215; *p* = 0.65) and no interaction (*F*_(1,22)_ = 0.001; *p* = 0.96). A similar pattern was observed for the eye tracking task, as there was a main effect of TRIAL (*F*_(1,22)_ = 8.65; *p* < 0.01; +17%), but there was no main effect of GROUP (*F*_(1,22)_ = 2.38; *p* = 0.14) or an interaction (*F*_(1,22)_ = 1.25; *p* = 0.27). The fact that after-effects had similar amplitude when being tested immediately after the removal of the perturbation, or when being tested after measuring after-effects in the other task rules out the possibility of strategic/explicit adaptation. Instead, our results are consistent with the view that, in both of our tasks, 40 trials of exposure to the visuomotor rotation induced sensorimotor adaptation.

### Supplementary analyses

Although evidence for an asymmetrical transfer between eye tracking and hand tracking is central to our objective, we felt the need to address possible confounds. First, we felt it was important to characterize gaze behavior during hand tracking. We found that while adapting to the rotation during hand tracking, both naive and experienced participants directed their gaze at the target, not at the cursor (i.e., rotated hand position). During the early stage of learning (first two trials), the eye–target distance was 3.5 times smaller than the eye–cursor distance (1.80 vs 6.31 cm; *p* < 0.001). Even during the last phase of adaptation (last two trials) in which cursor and target were closest to each other, gaze was still markedly closer to the target than the cursor (1.75 vs 2.83 cm; *p* < 0.001). Overall, this analysis suggests that the transfer of adaptation seen from hand tracking to eye tracking does not follow from gaze behavior given that, during hand tracking, gaze was poorly tied to the visual consequences of hand movement (i.e., cursor motion).

Second, we found that prior adaptation of hand tracking strongly influenced the accuracy of subsequent eye tracking, but was this effect corroborated by more intricate parameters of gaze behavior? To explore this issue, we examined the characteristics of catch-up saccades, a special type of saccade initiated to assist smooth pursuit when position and/or velocity error become too prominent (de Brouwer et al., 2002). When comparing the amplitude and rate of catch-up saccades during PRE and EARLY trials, we found in both cases a GROUP by TRIAL interaction (*F*_(1,22)_ > 7.65; *p* < 0.05). Breakdown of the interaction showed that for naive participants both the amplitude and rate of catch-up saccades increased by 28% when the rotation was introduced (*p* < 0.01), although no similar detrimental effect was observed for experienced participants (*p* > 0.17). Overall, these analyses confirm substantial improvements in gaze behavior following adaptation of hand tracking.

Third, we felt the need to ensure that the degree of randomness of target trajectories produced by naive and experienced participants during eye tracking was similar by means of ApEn (Pincus 1991; see also Landelle et al., 2016; Mathew et al., 2017). ANOVAs on target trajectory complexity showed no significant differences across groups for early (1–2), intermediate (19–20), and late (39–40) trials (*F*_(1,22)_ < 0.41; *p* > 0.52). As a result, we conclude that the higher accuracy of eye tracking exhibited by experienced participants does not stem from the fact that they performed fewer random hand movements than naive participants. Finally, using the same procedure, it was also found that the degree of target randomness was greater during eye tracking than during hand tracking (*F*_(1,22)_ > 15.75; *p* < 0.001). This observation rules out the possibility that the lack of transfer from eye tracking to hand tracking stems from the fact that participants were possibly exposed to more complex target trajectories during hand tracking than they previously were during eye tracking.

**Figure 7.**
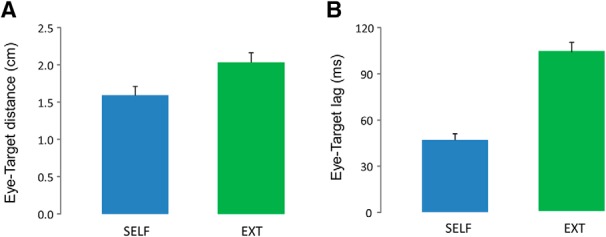
Comparison between eye tracking a self-moved target and an externally driven target. ***A***, Distance between eye and target. ***B***, Temporal lag between eye and target. Error bars represent the SEM. For both indices, eye tracking performance was more accurate during SELF.

Fourth, given the emphasis on predictive mechanisms in our eye tracking task, we felt it was crucial to provide baseline data regarding eye tracking performance when the target was no longer moved by the participant’s hand, but instead moved by an external agent. To explicitly address this issue, we ran a control experiment with 10 new participants that performed the original version of our eye tracking task (with no rotation), but also subsequently performed an eye tracking task in which their hand was immobile while we played back target trajectories they had generated when performing the previous task (Landelle et al., 2016; Danion et al., 2017; Mathew et al., 2017). The results presented in Figure 7 showed that, as expected, eye tracking performance was less accurate for playback trials than for those in which the target was self-moved. This view was confirmed by a one-way ANOVA showing a main effect of AGENCY such that during playback trials the eye–target distance increased by 24% (2.04° vs 1.68°; *F*_(1,9)_ = 8.9; *p* = 0.01; see Fig. 7*A*), and the eye–target lag doubled (104 vs 47 ms; *F*_(1,9)_ = 58.88; *p* < 0.001; Fig. 7*B*). Altogether, these results are consistent with the involvement of predictive mechanisms linking eye and hand actions when participants track a self-moved target. Finally, we observed that individual performance in each of these two tasks were uncorrelated. Indeed, the coefficient of correlation for eye–target distance, and eye–target lag were respectively 0.25 (*p* = 0.48) and 0.14 (*p* = 0.70). Based on these observations, we conclude that participants relied on separate control schemes to achieve these two types of eye tracking tasks.

## Discussion

Our main objective was to determine the extent of the coupling between the update of motor prediction and control when the mapping between hand motor commands and visual consequences is altered. To achieve this, we investigated the transfer of adaptation between a hand tracking and an eye tracking task both performed under visuomotor rotation. Our results showed an asymmetrical transfer such that prior adaptation with the rotation under hand tracking markedly improved eye tracking, but prior adaptation with the rotation under eye tracking did not benefit hand tracking. These findings have several implications relevant for prediction and control processes underlying hand movements.

A central assumption of the present protocol is that a forward model is updated during eye tracking. While this is supported by much work demonstrating that visuomotor adaptation is mediated by the reduction of visual prediction error generated by a forward model (Krakauer, 2009; Krakauer and Mazzoni, 2011), another possibility is that adaptation is due to model-free mechanisms that rely on reward prediction error (Izawa and Shadmehr, 2011; Haith and Krakauer, 2013). In such a scheme, a control policy could be improved through trial and error without the need of a forward model. Specifically, one could argue that, since in both tasks gaze was involved in tracking a moving target, improvements in the eye tracking task could follow from participants becoming better at tracking a moving target in general, which would then account for a transfer effect when switching from hand to eye tracking (given greater experience). Still, several observations make this possibility very unlikely. First, the fact that tracking a self-moved target and an externally moved target led to marked differences in terms of gaze behavior (Fig. 7), and that individual performances in these two tasks were uncorrelated, suggest that participants relied on different eye control policies to complete the eye- and hand tracking task. Second, if both tasks were to improve the same eye control policy, we should have observed some transfer of learning when switching from the eye-tracking task to the hand tracking task. In contrast, both groups exhibited similar gaze behavior during hand tracking. Namely, even during early trials of exposure, experienced participants did not exhibit a smaller eye–target distance compared with naïve participants. Third, when the rotation was introduced or removed, alterations in eye tracking were observed. Again, these alterations cannot be accounted for by an eye control policy that would simply improve eye tracking in general. Last, it has been shown that learning from sensory prediction errors is both faster and leads to a wider generalization function than learning from reward prediction errors (Izawa and Shadmehr, 2011; Haith and Krakauer, 2013). The fact that participants returned to baseline eye tracking performance within 10 trials and subsequently kept accurate eye tracking while still creating new hand trajectories demonstrates that this learning was rapid and generalized well (i.e., was not restricted to a single hand trajectory).

Following the adaptation of hand tracking, eye tracking performance under the rotation was immediately adequate (i.e., similar to baseline), which contrasts markedly with naive participants whose initial performance was clearly altered by the rotation. This near-complete transfer of adaptation is interpreted as evidence that, not only was the ability to generate hand motor commands to reach a desired cursor position updated, but so was the ability to predict visual consequences of hand movements. This observation fits well with the view that, at least for hand tracking, the update of both the inverse and forward model is mandatory for success. It is also consistent with computational work suggesting that force field and prism adaptation of reaching movements is best accounted for by a joint update of the forward and inverse models (Bhushan and Shadmehr, 1999; Honda et al., 2018), and that the update of a forward model is insufficient to produce optimal movements (Aprasoff and Donchin, 2012). Altogether, our study strongly suggests that to sustain accurate manual tracking under altered visual feedback, participants updated both the controller and the predictor of hand movements.

In contrast, hand tracking did not benefit from prior adaptation of eye tracking, as evidenced by similar poor performances in naive and experienced participants. We interpret this finding as evidence that, although participants were able to update their ability to predict visual consequences of hand movements, they did not update their ability to perform spatially directed hand movements. This observation supports the view that updating the neural mechanisms predicting the visual consequences of hand movement is not sufficient to subsequently control the cursor motion. With respect to internal models, this result can be taken as evidence that the forward model of hand movement can be updated in isolation of the inverse model.

To account for the present pattern of results, we propose that the update of an internal model is driven by task constraints, rather than by a systematic coupling between forward and inverse models. Indeed, in our eye tracking task, spatial constraints for hand movements were rather scarce, making the update of the inverse model unnecessary for success. We conclude that the update of control is task dependent and is achieved only if it is mandatory for the task. In contrast, the update of prediction was observed in both our tasks, suggesting that maintaining accurate sensory predictions of our movements is mandatory for efficient visuomotor adaptation. The fact that accurately predicting the consequences of our actions is key for many other brain functions, such as awareness of action, sensory cancellation, motor imagery, and social cognition (Wolpert and Flanagan, 2001; Schubotz, 2007; Bubic et al., 2010; O’Reilly et al., 2013; Kilteni et al., 2018), has perhaps also encouraged this update.

Experimental and computational work has already proposed that the update of prediction and control can exhibit different dynamics, the update of prediction being significantly faster than the update of control (Bhushan and Shadmehr, 1999; Flanagan et al., 2003; Yavari et al., 2013). This scheme is supported by our study in which adaptation was found to be faster and more complete during eye tracking (return to baseline within 10 trials only) compared with hand tracking (no return to baseline after 40 trials). One possible reason for this discrepancy is that the update of an inverse model is computationally more demanding than the update of a forward model (Jordan and Rumelhart, 1992; Miall and Wolpert, 1996; Wolpert and Kawato, 1998). Indeed, although many motor commands can potentially provide the same desired output (i.e., for redundancy problem, see Bernstein, 1967), a motor command is unambiguously linked to a particular sensory feedback. It is of interest to note that asymmetrical transfer has been previously reported in the context of visuomotor adaptation (Morton and Bastian, 2004; Krakauer et al., 2006; Wang and Sainburg, 2006). In particular, it has been shown that during exposure to a 30° visuomotor rotation, arm training benefited subsequent wrist training, but not vice versa (Krakauer et al., 2006). Moreover, the adaptation of wrist movements was markedly faster than the adaptation of arm movements. Altogether, this study and the current one suggest that there might be a tradeoff between the speed of visuomotor adaptation and the flexibility for generalizing this adaptation to other contexts.

In most experiments that investigate sensorimotor adaptation of arm movements, it is challenging to dissociate the influence of forward and inverse models (Lalazar and Vaadia, 2008; Mulliken et al., 2008), because, as suggested by our study and others, both contribute to adaptation but do so in different ways (Honda et al., 2018). However, the present design combining hand- and eye-tracking movements allowed us to unpack these two contributions and to isolate the update of the forward model. Further studies will have to explore whether our findings, obtained through adaptation to visuomotor rotation, extend to prismatic adaptation and/or force field adaptation, two other key paradigms used to investigate the update of internal models (Shadmehr, 2004, 2017; Petitet et al., 2018). Note that this is not necessarily the case, as for prismatic adaptation it has been shown that the viewing of active (but not passive) rhythmic arm movement with no explicit target leads to subsequent adaptation of discrete arm movements toward explicit targets (Held and Freedman, 1963; see also Held and Gottlieb, 1958). However, it is not straightforward to circumvent the origin of a discrepancy between our current observations and this previous finding. First, as pointed out recently, the transfer profile of prism adaptation contrasts in several ways with other adaptation paradigms (including visuomotor rotation), which thereby requires special attention for this experimental model of sensorimotor integration (Petitet et al., 2018). Second, unfortunately gaze analysis as well as possible instructions given to the participants regarding gaze behavior were not included in those early prismatic adaptation studies (Held and Gottlieb, 1958; Held and Freedman, 1963).

In general anticipatory control, as evidenced when eye tracking a self-moved target (Scarchilli et al., 1999; Vercher et al., 2003), manipulating objects (Flanagan and Wing, 1997; Danion and Sarlegna, 2007) or coordinating several effectors (Diedrichsen et al., 2007) is often taken as evidence of forward models that predict the consequences of an upcoming action (Miall and Wolpert, 1996; Wolpert et al., 2011), but alternatively, anticipatory control can reflect a feature of a good control policy that was learned via model-free or model-based mechanisms (Haith and Krakauer, 2013). Although additional experiments are needed to tease these two options apart, within the framework of internal models, our study challenges the view that forward and inverse models are coupled during their acquisition (Wolpert and Kawato, 1998; Kawato, 1999; Haruno et al., 2001; Honda et al., 2018) and suggests a more flexible relationship between the two. Not only would this confirm that the update of forward and inverse models can exhibit different dynamics (Bhushan and Shadmehr, 1999; Flanagan et al., 2003; Honda et al., 2018), but we propose that the forward model can be updated independent of the inverse model. More generally, our study promotes the view that prediction and control are mediated by separate neural processes (Shadmehr et al., 2010; Scott, 2012), and suggests that people can learn to predict movement consequences, without necessarily promoting their ability to control these movements. Finally, it has been demonstrated recently that task demands are critical for the update of sensory predictions (Auksztulewicz et al., 2017), and the current study extends this notion to the update of movement control.

## Synthesis

Reviewing Editor: Trevor Drew, University of Montreal Decisions are customarily a result of the Reviewing Editor and the peer reviewers coming together and discussing their recommendations until a consensus is reached. When revisions are invited, a fact-based synthesis statement explaining their decision and outlining what is needed to prepare a revision will be listed below. The following reviewer(s) agreed to reveal their identity: R. Christopher Miall.

Reviewer#1

The authors try to dissociate prediction and control in motor adaptation, presenting data suggesting an asymmetrical relationship between the two. Specifically, they examine potential transfer of adaptation between two tasks: (a) when hand adapts to a visuomotor rotation during a tracking task and (b) where the eyes are required to track the movement of a cursor which is the result of self-generated random hand motion under a similar visuomotor rotation. They find that hand adaptation facilitates eye tracking but not vice versa. As the authors assume that the hand tracking task would reflect adaptation of both prediction and control but the eye tracking task would reflect updating of prediction only, they deduce that prediction and control are separate processes, and that updating a predictor for the sensory outcome of a movement doesn't necessarily update the ability to control that movement.

This perspective is of particular interest to the motor control community, especially in light of the widespread perception that adaptation occurs when the forward model, updated by sensory prediction error, then trains the inverse model (“inversion” of the forward model). Thus, such work could be both useful and timely.

However, the authors' interpretation hinges entirely upon the assertion that improvement on the eye-tracking task occurs via a forward model. The evidence for this, however, is not convincing. Improvements in eye-tracking under the rotated environment need not reflect the update of a predictive forward model; they could just reflect improvements in a control policy for the eye, which could have been learned through model-free mechanisms, e.g. trial and error, without the need to go through a forward model. The motor system might be better at anticipating (through better tuned control policies) but not necessarily at predicting (through a forward model). Anticipation does not equal prediction (see Haith and Krakauer 2013). In other words, the subject might be becoming better at tracking a target with their eyes (better under a rotation, or even just better at tracking a moving target in general). When transferring from the hand tracking to the eye-tracking task, participants might benefit because they practiced eye tracking of the target during the hand tracking task. When transferring from eye-tracking to hand tracking, improvements in eye tracking do not additionally help hand tracking.

Thus, for the paper's main point to stand, the authors would need to better demonstrate that a forward model of the hand is indeed being updated in the eye tracking task. The paper does not show this, and coming up with an experiment to prove it will be, unfortunately, not an easy task.

A few ideas:

• If participants are simply benefitting from eye tracking practice, then the benefits of transfer from hand-tracking to eye-tracking would be similar even if the visuomotor transformations imposed in each case were of a different kind - e.g. transfer from a rotation to a shear or vice versa.

• I wonder if the eye vs. hand tracking data could offer some insight: are the eyes following the hand (more in line with eye position being a readout of a forward model), or is the hand following the eye (more in line with eye position indicating the target of hand movement / goal of the control policy - in the sense that, for example, gaze can tell us about where participants are aiming in a reaching task (deBrouwer et al., 2018).

• They could have the subjects subsequently track the cursor with their left hand rather than their eye - if it is a forward model it should generalize whereas an eye-specific policy might be specific.

• Check for change in perceived position as was done by Izawa and Shadmehr (2011).

Even assuming that the eye-tracking task indeed amounts to the training of a forward model, there might be other interpretations to the data. Implicit adaptation can be heavily context-specific and tied to specific motor plans (even if the actual movements might be similar, e.g. (Howard et al., 2015) and others along the same lines), with transfer to other contexts severely limited.

• When transferring from the eye-tracking task to the hand-tracking task, the goals and motor plans are drastically different: participants move from essentially random movement to specified motor plans which are in all likelihood much different than the ones the participant went for during eye-tracking. Thus, even if the forward model were indeed in general able to train the inverse model, this wouldn't be expressed here, since the original adaptation of the forward model during eye-tracking is assigned to a much different context.

• In contrast, when transferring from the hand-tracking to the eye-tracking task, participants might have used (at least at the first 4-5 trials - that's where the differences lie between the curves in Figure 6B) plans similar to the ones they were trained on during the previous hand-tracking task. The authors use some metrics to argue that these movements are indeed random; I also understand this analysis used the whole block. I was wondering if the random movements during eye-tracking, especially for the first 4-5 trials, are more similar to the hand-tracking movement patterns in the group where the hand-tracking task came first.

• During the hand tracking task, participants focus their gaze on the target (primarily), but during eye-tracking, they have to focus their gaze on the cursor. These are different contexts/goals, and that should further limit transfer. At the end of the hand-tracking task, participants gaze might tend to get aligned with the cursor (since gaze generally reflects the target, and with learning the cursor better follows the target), meaning transfer to the cursor-specific eye-tracking task might be easier.

Overall, I am very a sympathetic to the project and the results are interesting. They may even end up being attributable to what the authors claim. That said, they commit the regrettably all too common mistake of equating anticipation with forward-model-based prediction. A more direct demonstration of a forward model, or at least a step in that direction, is needed. Alternatively, at the very least a more modest framing of the paper with a more ecumenical admission that other interpretations are possible.

Minor:

Line 67: online corrections by the inverse model?

Line 69: “and should be associated...” do the authors mean that producing such optimal movements should instead be associated with the update of the controller?

Line 178: in -&gt; of

Line 219: is required

Line 445: date missing from the citation

Line 454: The reasoning behind relating results such as (Krakauer et al., 2006) to the authors' is not that clear, It needs to be better fleshed out.

Reviewer #2

This paper presents the results of a single, straightforward experiment testing the generalisation of learning to predict hand movement between two tasks, one with the goal to track an external target, using eye and hand movement, and one with the goal to track the hand movement with eye movement. An additional control group tested tracking of external movement with the eyes alone. The authors use the results to show quite convincingly that there can be separation of the update of predictions about hand movement (as a target for eye movement) independent of the control of hand movement (using prediction of hand-to-cursor mapping).

There are two main issues with the overall context of the paper. First, it is something of a straw man to argue that prediction and control are obligatorily related. One early scheme (Miall's Smith Predictor) had this property, since it achieved “inverse modelling” by placing the forward model in a feedback loop, but most theories now have explicit and separate forward and inverse models. So the authors need to make a stronger argument (or drop the argument) that these are obligatorily linked.

Second, their results demonstrate that the condition of eye tracking the hand does not generalise to hand tracking, and the implication of course is that the is no update of the inverse model. This is somewhat surprising as an analogous task - prism adaption without explicit targeted hand actions - does lead to adaption of the arm movements. This discrepancy should be discussed further.

The analysis of the control experiment showing eye-target lag is also good, and this analysis of lags should be included for both of the main experiments (eye-cursor separate from eye-target for hand tracking, and potentially both eye-hand and eye-cursor for the eye-tracking task). It may reveal interesting change in the eye-tracking performance during the hand-tracking task.

Additional comments:

The analysis of the two tasks is different, and should be similar, based on exponential curve fits (the eye data are fitted by linear regression over 10 trials).

The interleaving of alternate X and Y data is a strange thing to do, as the alternation will very strongly drive the cross-correlation (I think, but am not certain, this is particularly likely if the X and Y data are not independent). Please justify, and consider taking the average of separate correlations of X and Y signals.

The test of trajectory complexity is good, but it appears restricted to just 10 trials (line 370). Why?

Minor comments:

Line 312 - which 5 other measures? Do you mean trials (the 3 “performance measures” are the averages of trial pairs)?

Line 322 and 411 - transfer is not complete - there is a noticeable uplift from baseline and a gradual return to baseline across the whole of the session. “Near-complete” would be acceptable.

Lin 337 - one could take issue with whether a pair of POST trials is sufficient to exclude strategic effects: the first of these trials will be a surprise trial and would retain whatever strategy was used at the end of adaption, the second trial may or may not have strategic responses to this error.

Line 354 - the term “too important” is odd, replace with “too prominent
